# Acknowledgement to Reviewers of *IJERPH* in 2015

**DOI:** 10.3390/ijerph13010150

**Published:** 2016-01-22

**Authors:** IJERPH Editorial Office

**Affiliations:** MDPI AG, Klybeckstrasse 64, CH-4057 Basel, Switzerland; ijerph@mdpi.com

The editors of *IJERPH* would like to express their sincere gratitude to the following reviewers for assessing manuscripts in 2015. 

We greatly appreciate the contribution of expert reviewers, which is crucial to the journal’s editorial decision-making process. Several steps have been taken in 2015 to thank and acknowledge reviewers. Good, timely reviews are rewarded with a discount off their next MDPI publication. By creating an account on the submission system, reviewers can access details of their past reviews, see the comments of other reviewers, and download a letter of acknowledgement for their records. We are grateful to our reviewers for the contribution they make.

A. Fuller, James
Aarts, Ronald M.
Aasvang, Gunn
Abbott, Louise
Abdelrahman, H. M.
Abdul Hamid, Zariyantey
Abe, Kaoru
Abrahão, Renata
Abson, David J.
Abson, Dave
Abughosh, Susan M.
Ackermann-Liebrich, Ursula
Acosta Jr., Frank L.
Acuna-Soto, Rudofo
Adams, Alex
Adams, Rachel I.
Adedimeji, Adebola
Adirim, Terry
Aerts, Sam
Agarwal, Priyanka
Agbemabiese, Lawrence
Agodi, Antonella
Agostoni, Piergiuseppe
Aguilar-Luzón, María del Carmen
Agusa, Tetsuro
Ahiablame, Laurent
Ahmed, Warish
Ahmed, Khalil
Ahn, Sukhee
Ahn, Sun Joo (Grace)
Ahn, Kwangmi
Aittasalo, Minna
Ajani, Penelope
Akin, Su-Mia
Al-Qodah, Zakaria
Al-Taiar, Abdullah
Alatrista-Salas, H.
Alavanja, Michael
Albini, Angelo
Albiol, Sergio
Alekseenko, Vladimir
Alessandri, Guido
Alexander, Kimberly E.
Alexander, Vaninsky
Alfonso, Moya
Ali, Khalid Mustafa
Ali, Saleem
Allebeck, Peter
Allen, Michael J.
Allende Prieto, Ana
Allison, Susannah
Almadi, Tawfiq
Almeida, Susana Marta
Almoznino, Galit
Altenburg, Teatske
Althouse, Benjamin M.
Álvarez, Silvia Izquierdo
Alvarsson, Jesper
Amarasena, Najith
Amenta, Francesco
Amerson, Roxanne
Amir, Lisa
Amy R., Wilson
An, Xingqin
An, Ruopeng
Analitis, Antonis
Andersen, Helle R.
Anderson, Anne J.
Anderson, Rhona M.
Andjelkovic, Mirjana
Andrade, Fernando
Andrade, Leonardo N.
Andreeva, Tatiana
Andrejčáková, Zuzana
Andreotti, Gabriella
Andrew, Sussman
Aneziris, O.N.
Angelici, Maria Cristina
Angell, Yvonne
Angulo, Elsa Delgado
Anido Rifón, Luis E.
Ansai, Toshihiro
Ansari, Mohd Ikram
Anselmi, Luciana
Anson, Yonathan (Jon)
Anstey, Kaarin J.
Antikainen, Harri
Apekey, Tanefa
Aplin, Tammy
Appleton, Sarah
Applewhite, Steven
Apsley, Andrew
Aradi, Renata
Arantegui, Marta Rusiñol
Araujo, Rosa
Arbona, Vicent
Arbuckle, Tye
Arbuthnott, Katherine
Arcidiacono, Biagio
Argikar, Upendra
Argos, Maria
Armanini, Decio
Aronow, Wilbert
Arslan, Seza
Aruguete, Deborah
Arugula, Mary
Asakawa, Akihiro
Asakura, Hiroshi
Asakura, Takashi
Ashour, Mohamed-Bassem
Asimakopoulos, Alexandros G
Askew, Eldon Wayne
Aslan, Asli
Assani, Ali A.
Assari, Shervin
Astell-Burt, Thomas
Auger, Nathalie
Augustijn, Denie
Aurrekoetxea, Juan J.
Austin, Stephanie E.
Averett, Susan L.
Avissar, Sofia
Aw, Tiong Gim
Axelsson, Gösta
Ay, Murat
Ayres, Cynthia
Azagba, Sunday
Azorin, Jean-Michel
Baba, Sachiko
Babajko, Sylvie
Bach, Horacio
Bachou, Hanifa
Bachschmid, Markus M.
Badariene, Jolita
Bae, Jong-Myon
Bagby, Michael
Bagley, Erika J.
Bagnara, Maurizio
Bahle-Lampe, Angela
Baigorri, Roberto
Bailey, Craig B.
Bainbridge, Daryl
Baird, Ian
Baker, Elizabeth A.
Bakhireva, Ludmila N.
Balachova, Tatiana N.
Baláž, Matej
Balazs, Judith
Balbi, G.
Balcazar, Hector
Ballester, Ferran
Balocco, Carla
Bambach, Mike
Bambrick, Hilary
Bamidis, Panagiotis
Bande, Roberto
Banerjee, Areen
Banik, Gouri Rani
Banks, Jonathan
Bansal, Shweta
Bao, Mutai
Bapputty, Reena
Bar-On, Elhanan
Barba-Brioso, Cinta
Barbosa-Leiker, Celestina
Bares, Cristina
Barnard, Yvonne
Barnett, Lisa M.
Barregård, Lars
Barrett, Emily S.
Barrios, Rocío
Barroso-Bogeat, Adrián
Bartolucci, Giovanni Battista
Bartram, Jamie
Barzyk, Timothy M
Bashir, Shahid
Bastian, Olaf
Batanova, Milena
Batista, Bruno Lemos
Batista, Cristina
Batley, Graeme
Batterham, Philip J.
Baum, Carl R.
Baxter, Peter
Baxter, Lisa
Bearman, Chris
Beaver, John
Bednarska, Agnieszka J.
Bedri, Zeinab
Beedie, Chris
Been, Jasper V
Beil, Kurt
Bell, Simon
Bell, Sarah L.
Bell, Lynne
Bellasio, Roberto
Bellia, Laura
Belviso, Claudia
Belyaev, Igor
Ben-Dov, Iddo Z.
Ben-Natan, Merav
Benfield, Jacob
Benitez, Gabriela Vazquez
Benjankar, Rohan
Benmarhnia, Tarik
Bennett, Kate M
Bennett, Derrick A.
Bennett, Simon J
Benowitz, Neal
Bensch, Gunther
Berardi, Umberto
Berardinelli, Francesco
Berbel, Pere
Bere, Elling
Bergamaschi, Enrico
Berkvens, Dirk
Berman, Jesse
Berman, Tamar
Bermejo-Nogales, Azucena
Bernardo-Bricker, Anna
Bernat, Przemysław
Bernaudat, Ludovic
Bernert, John
Berns, Monika
Berrigan, David
Berry, Peter
Bert, Fabrizio
Berthelot, Philippe
Bertoldo, Raquel Bohn
Bertolotti, Gian Mario
Bertrand, Martine
Berzano, Marco
Besalú, Emili
Bessa, Lucinda
Bessell, Paul R
Bethea, Jane
Bethel, Jeffrey
Beutell, Nicholas J.
Beverland, Iain
Beyer, Kirsten
Bhandari, Gokul
Bhandari, Medini
Biales, Adam
Bianchi, Renzo
Biavasco, Francesca
Bierer, Barbara E.
Bilancia, Massimo
Bilgili, Özge
Billington, Rex
Bilotta, Patrícia
Binns, Colin
Biron, David G.
Biron, Caroline
Birzer, Cristian H.
Bisharat, Naiel
Biziuk, Marek
Bjerregaard, Peter
Bjørklund, Geir
Blackmore, Mark
Blackstone, Robin
Bláha, Ladislav
Blair, Pete
Blanco, Angeles
Blázquez, Maribel
Bloch, Joan Rosen
Blondeau, Joseph
Bloom, David E.
Bloomer, Steven
Blum, Jason L.
BOCCIO, DANA E
Bock, Michael
Boeckmann, Melanie
Boehmer, Tegan
Boen, Filip
Boffi, Roberto
Bogen, Kenneth T.
Bogin, Barry
Bogosian, Angeliki
Boischio, Ana
Bojorquez, Ietza
Bolte, John
Bolton Moore, Carolyn
Bond, Virginia
Bonetta, Silvia
Bonnet, Pierre A.
Bonsall, David
Bonuck, Karen
Booth, Josie
Boraschi, Diana
Borde, Theda
Borgnakke, Wenche S.
Borisenkov, Mikhail F.
Borja, Judith B
Börjesson, Stefan
Borowski, Jacek
Boscoe, Francis P.
Botte, Sandra
Bottone, Maria Grazia
Bouaïcha, Noureddine
Bouckenooghe, Dave
Bousse, Tatiana L.
Bovee, Toine F.H.
Bowen, Nathan
Bowling, Lee
Boyas, Javier F.
Boyd, Robert S.
Boyer, Treavor
Boyland, Emma
Braca, Mauro
Brackbill, Robert M.
Bradshaw, Tim
Bragge, Peter
Brand, Larry
Brandl, Alexander
Brandon, David
Brandón, María Gómez
Brandt, Diane E.
Brasso, Rebecka
Bråtveit, Magne
Braune, Birgit
Bravaccio, Carmela
Breitner, Susanne
Brevik, Eric C.
Bricard, Damien
Briggs, David
Briner, Wayne
Brink, LuAnn L.
Brink, Mark
Brink, Yolandi
Briones, Rowena L.
Brites, Alice
Brito, Luísa
Broadhurst, Jennifer
Brodsky, Bs
Bron, Anthony
Broniatowski, David Andre
Brook, Libby
Broome, Kieran
Brosseau, Lisa M
Brown, David
Brown, Greg
Browning, Matthew
Brucker, Debra L
Bruckner, Tim A.
Brun, Nadj
Brunetti, Gennaro
Brunetto, Yvonne
Brymer, Eric
Bryne, Patrick
Brzozowska, Renata
Buchanan, Robert L.
Buchanich, Jeanine M.
Bucio, José López
Buck, David
Buckey, Jay
Buckley, Timothy
Buja, Alessandra
Bujnowska-Fedak, Maria Magdalena
Bukrinsky, Michael
Bull, Sheana S.
Bullen, Chris
Bullock, Russ
Bullon, Pedro
Bunch, Martin J.
Burgess, John A.
Burgess, Jeff
Bürgi, Alfred
Burkart, Katrin
Burns, Brendan
Burrowes, Jerrilynn
Burstyn, Igor
Burtscher, Martin
Bus, James S.
Buscemi, Joanna
Butler, Stephanie
Butt, Talib E.
Buunk-Werkhoven, Yvonne A. B.
Buxton, Jane
Byrne, Hugh J.
Byun, Wonwoo
Caan, Woody
Cabello, Maria
Cable, Noriko
Cabral, João P. S.
Cacace, Mirella
Cadwell, Karin
Cai, Qiang
Calderón-Garcidueñas, Lilian
Caldwell, Bruce
Caldwell, Brent
Calistri, Paolo
Camarda, Giancarlo
Cameron, Debra
Camoni, Laura
Campagna, Marcello
Campbell, Michael J.
Cancio, Ibon
Candeias, Carla
Caneday, Lowell
Cao, Yang
Cao, Yinzhi
Cao, Chunxiang
Capilouto, Gilson
Capuani, Silvia
Carden, Kirsty
Cardenas, Christian L. Lino
Carey, Mark
Carignan, Courtney C
Carling, Philip C.
Carling, Greg
Carlsen, Hanne Krage
Carney, Jeffrey
Carney, Timothy Jay
Caron, François
Carpenter, David
Carratalà, Anna
Carreira Merquior, Vânia Lúcia
Carrero, Jose Antonio
Carson, Arch
Carter, Sylvester
Carter, Ellison M.
Caruso, Joseph A.
Casale, Marisa
Case, Bruce
Cash, Scottye
Castaño-Corsino, Zafira
Castillo, Francisco Javier
Castillo-Rodal, Antonia Isabel
Castro-Schilo, Laura
Catalanotti, Jillian S.
Cataldi, T.R.I.
Cato, Michael
Cauchie, Henry-Michel
Caulfield, Brian
Cauter, Eve Van
Centeri, Csaba
Cermak, R.
Cervera, Richard
Cervero Aragó, Sílvia
Cesari, Daniela
Ceuppens, Siele
Chadwick, Elizabeth A
Chai, Yanwei
Chaiton, Michael
Chakraborty, Mukta
Chalifour, Lorraine
Chalkias, Christos
Chambel, Maria José
Chambers, Catrina
Chan, Polin
Chan, Fong
Chan, Christian
Chan, Ta-Chien
Chan, Christopher
Chan, Catherine
Chan, Mark
Chan, P. W.
Chan, Derwin King-Chung
Chan, Siew Pang
Chan, Thomas
Chan, King Ming
Chan, Ying-Chieh
Chandler, Cynthia Kay
Chang, Li-Chun
Chang, Howard
Chang, Seo Il
Chang, Chun-Yen
Chang, Chih-ching
Chang, Shun-Jen
Chang, Hoshing
Chang, Shu-sen
Chang, Chih-Jen
Chang, Wen-Pin
Chang, Ta-Yuan
Chang, Kee-lung
Chaparro, M. Pia
Chapman, Simon
Charisiadis, Pantelis
Charles, Luenda E.
Charlson, Fiona
Charnock, Colin
Charvalos, Ekatherina
Chatterji, Somnath
Chatzidiakou, L.
Chau, Kwok-Wing
Chaves, Luis Fernando
Cheah, Nuan Ping
Chen, Ku-fan
Chen, Zhijun
Chen, Peijie
Chen, Kow-Tong
Chen, Wei-Hong
Chen, Dongmei
Chen, Chien-yuan
Chen, ZhiQiang
Chen, Xiang (Peter)
Chen, Ziqi
Chen , Zueng-Sang
Chen, Dingjiang
Chen, Xiaoguang
Chen, Xi
Chen, Yen-Chih
Chen, Tao
Chen, Chun
Chen, Ting-chien
Chen, Jie
Chen, Jianjun
Chen, Xinguang
Chen, Ruey-Yu
Chen, Chieh-fu
Chen, Ray
Chen, Cherry
Chen, Yi-Chun
Chen, Chien-Fu
Chen, Gau-Yang
Chen, Jung-Wei
Cheng, Chih-hsiu
Cheong, Hae-kwan
Chernoff, Neil
Chernyak, Sergei
Cherukupalli, Rajeev
Cheruvu, Vinay K.
Cheung, Wing-Hoi
Cheung, Chau-kiu
Cheung, Teris
Chiang, Li-Chi
Chilibeck, Phil
Chillón, Palma
CHIODINI, Paolo
Chiou, Tzeon-Jye
Chipps, Jennifer
Chitnis, Nakul
Cho, Arthur Kenji
Choi, Jeong-Hee
Choi, Ching
Choi, Kyungho
Choi, Shin Sik
Choi, Young-Kwan
Choi, Seo-Eun
Choi, Doo-Sup
Chong, Marc
Choppala, Girish
Chou, Yu-Ching
Chou, Wen-ying Sylvia
Chousalkar, Kapil
Chow, Sin Yin Alice
Chowell, Gerardo
Christensen, Henrik
Christensen, Morten Lykkegaard
Christophe, Daniel
Christophe, Waterlot
Chruszcz, Maksymilian
Chrysikopoulos, Constantinos V.
Chrzanowski, Łukasz
Chuang, Kai-Jen
Chudal, Roshan
Chun, Kwok Pan
Chung, Tammy
Chung, Min-Huey
Ciarkowska, Krystyna
Ciccone, Marco Matteo
Cieszynska, Monika
Cigler, Beverly A.
Cincinelli, Alessandra
Cipolletta, Sabrina
Cizmas, Leslie
Claassen, Liesbeth
Claeson, Anna-Sara
Clarke, Nick
Clarke, Helen
Clarren, Sterling
Clarys, Peter
Clasen, Thomas
Clayton, Susan
Cledon, Maximiliano
Cleland, John G.f.
Clemente, Rafael
Clements, C. John
Clifton, Judith
Clifton, Peter
Cloutier, Scott
Clover, A. James P.
Cochran, Lessie L.
Cochrane, Tom
Coe, Christopher L.
Cohen, Marc
Cohen, Chari
Cohen, Steven A.
Colagiuri, Ruth
Coleman, David
Coleman, Tim
Collado, Silvia
Collavin, Licio
Collie, Rebecca
Collier, Sarah A.
Collings, Paul
Colombo, Giancarlo
Colon-Otero, Gerardo
Coloso, Claudio
Comasco, Erika
Comi, Antonio
Conceição, Eusébio Z.E.
Congestri, Roberta
Conklin, Daniel
Connolly, Deirdre
Contini, Daniele
Contino, Francesco
Cooke, Marcus
Cornell, Morna
Cornett, Jack
Cornish, Stephen
Cornman, Deborah
Cornu, Catherine
Correia-Deur, Joya
Correll, Ray
Corsaro, Daniele
Cortes, Jorge Cerna
Costa, Silvia
Costa, Silvia Figueiredo
Costa, e Inês
Costa-Rodrigues, João
Coughenour, Courtney
Coupland, L. J.
Cousins, Rosanna
Craig, Eva
Craig, Jennifer P
Craig, Laura
Crary, Michael
Crawford-Brown, Douglas J.
Crews, John E.
Crichton, Fiona
Crone, Diane
Cronkite, Ruth C.
Crossley, Alison
Cruise, Hanker
Cruz Campas, Martín Eusebio
Cservenka, Anita
Cuberek, Roman
Cui, Jian
Cui, Lixian
Cullinan, Paul
Culp, Ken
Cummings, K. Michael
Cunningham, Kenda
Curtis, Val
Cyrys, Josef
D'Agatta, Elisabetta
Da Cunha, Kenya Moore Dias
Da'na, Enshirah
Dabek-Zlotorzynska, Ewa
Dahiya, Anju
Dailianis, Stefanos
Daker-White, Gavin
Dakubo, Crescenti Y.
Dakulincová, Zuzana
Dall, Philippa
Dallimer, Martin
Damalas, Christos
Dancer, Stephanie
Daniele, Michael A.
Danuso, Francesco
Dargan, Paul
Dassenakis, Μ anos J.
Dautzenberg, Betrand
David, Jamil
Davídkovová, Hana
Davidson, Larry
Davies, Hugh
Davies, Anita A.
Davis, Claudia M.
Davis, Melissa
Dawes, Daniel E.
Dawes, James
Dawkins, Lynne
Dawson, Kenneth A.
Day, Carolyn
Daye, Desalegn Desissa
De Boer, Michiel P.
de Castro, Shamyr Sullivan
De Castro Nery, Susana Vaz Da Silva
de Donato, Francesca
De Jong, Elske
De Kinderen, Reina
De Kluizenaar, Yvonne
De Lourdes Pereira, Maria
De Paiva, Cintia S.
De Perio, Marie
De Ridder, Koen
de Santiago-Martín, Ana
De Sayu, Rebecca Paradiso
De Seze, Rene
De Vocht, Frank
De-Witt Huberts, Jessica De-Witt
Dean, Sheila
Debattista, Joe
Debes, Amanda K.
Debost-Legrand, Anne
Decker, Brooke
Defreese, J.D.
DeHart, Dana
Deka, Devajyoti
Del Hoyo, C.
Dela Cruz, Felicitas A.
DeLisi, Matt
Delisle, Hélène
Delmelle, Elizabeth C.
Delmelle, Eric M.
Deng, Bing
Deng, Wenfeng
Dennis, Sarah M.
Denny, Clark
Denton, Sarah
Depcik, Christopher
Déri, Andrea
Derman, Peter
Derose, Kathryn Pitkin
DeRouen, Mindy C.
DeSesso, John M.
Deurwaerdère, Philippe
Devine, Carol M.
Dhar, Ratan
Di Guardo, Antonio
Di Martino, Ferdinando
Diaz, Julio
Diaz, Theresa
Diaz, Alejandro
Dickens, Joseph
Dickerson, Aisha S.
Dickson-Swift, Virginia A
Diebo, Bassel G.
Dietz, Noella
DiGiacomo, Michelle
Dijk, J.P. (Jitse) van
Dilley, Julia A.
Dimitriou, Elias
Dimitrov, Borislav
Dinas, Petros
Ding, Wenjun
Dingle, Genevieve
Dinu, Cerasela Zoica
Diringer, Sarah
Dirks, Kim Natasha
Djebarni, Ramdane
Dobbs, Cynnamon
Dodd, Peter J
Doerschuk, Claire M
Doherty, Thomas
Dohi, Yasuaki
Dollman, James
Dolphens, Mieke
Domingo, Rosario
Domínguez-Reyes, Antonia
Donath, Lars
Donatuto, Jamie
Dong, Ming
Dong, Zhao
Dong, Morrow
Donne, Bernard
Donzelli, Sabrina
Dorea, Jose
Dortet, Laurent
Dost, Turhan
Doudrick, Kyle
Dove, Melanie
Doyle, Glenn A.
Drahota, Petr
Dreibelbis, Robert
Drenowatz, Clemens
Drouiche, Nadjib
Drydakis, Nick
Du Prel, Jean-Baptist
Duan, Weili
Duan, Wenjie
Duann, Jeng-Ren (JR)
Duarte, Elizabeth
Dubovsky, Steven L.
Duclot, Florian
Duer, Wolfgang
Dumas, Orianne
Dumitrascu, Dan
Dummer, Trevor
Dumont, Patrick
Dunlap, Kriya
Dunn, Kelly
Durek, Julia
Durkin, Anne
Durkin, Helen
Dürrenberger, Gregor
Dusso, Adriana
Duty, S. M.
Dworkin, Mark
Dyche, Jeff
Dziubanek, Grzegorz
Eames, Catrin
Earles, Julie L.
Earthy, Jonathan
Eastman, C.I.
Ebi, Kristie
Eck, William S.
Eder, Milton Mickey
Edo, Andrew
Edwards, Roger
Edwards, Joshua R.
Edwards, Christine A
Efird, Jimmy
Eggo, Rosalind
Eglash, Anne
Egnoto, Michael J.
Eiche, Elisabeth
Eime, Rochelle M
Eisenberg, Marlene
Ekenga, Christine C.
Ekuni, Daisuke
Ekwall, Daniel
El Muayed, Malek
El-Geneidy, Ahmed
Elam-Evans, Laurie D.
Elia, Antonia Concetta
Elliott, Susan
Ellis, Kate
Elmenhorst, Eva-Maria
Elsharafi, Mahmoud
Elstad, Jon Ivar
Emerson, Eric
Enders, Achim
Engelen, Lina
Engelman, Alina
English, Paul
Enki Yoo, Eun-Hye
Enoch, Mary-Anne
Ensoli, Barbara
Erazo, Marcia
Erdal, Serap
Erdely, Aaron
Erikson, Keith
Eriksson, Eva
ERNESTO, SALZANO
Ervin, Kaye
Escario, José-Julián
Esmi, Caue Ferreira
Esposito, Ciro
Esser, André
Esser, Ingrid
Esteban, María Esther
Esteban, Irene
Esteve-Cano, V.
Estrada-Garcia, Teresa
Ettema, Dick
Etter, Jean-Francois
Ettler, Vojtech
Etzel, Ruth
Evans, Richard
Everingham, Jo-Anne
Ewa, Bok
Exadaktylos, Aristomenis
Eyrolle-Boyer, Frédérique
Faas , Dolmans L. Servaas
Fabbricino, Massimiliano
Factor-Litvak, Pam R.
Fagerstrom, Karl
Fairfield, Charlie
Falkinham, Joseph O.
Fallacara, Dawn M.
Fan, Yingling
Fan-Hagenstein, Haiyan
Fang, Tao
Fantke, Peter
Farber, Harold J.
Farkas, Csilla
Farma, Jeffrey M.
Farr-Wharton, Rod
Farsallinos, Konstantinos
Faruqi, Nighat
Farzan, Shohreh
Fassò, Alessandro
Fattore, Elena
Fatyeyeva, Kateryna
Faulhaber, Martin
Favero, Gabriele
Faxelid, Elisabeth
Feinkohl, Insa
Feng, Zhanchun
Feng, Zhiqiang
Ferguson, Sue A.
Fergusson, David M.
Ferketich, Amy
Fernández, Carlos Sierra
Fernandez, Blanca
Fernandez, Marina
Fernández, J. A.
Fernandez, Maria Luz
Fernando, Eustace
Fernando, Ana Luisa
Ferrante, Margherita
Ferrara, Santo Davide
Ferrari, Nina
Ferraz, A. I.
Ferré, Georgina Guilera
Ferris, William
Fichera, Eleonora
Fiechtner, Lauren
Figueroa, Wanda
Figueroa Meza, Maria Josefina
Filia, Antonietta
Finazzi, Guido
Finazzi, Francesco
Findlay, Leanne C.
Findorff, Mary
Fine, James Burke
Finkelman, Robert B.
Fitzgerald, Edward
Fitzpatrick, Patricia
Flanagan, Sara V.
Flander, Louisa
Fledderjohann, Jasmine
Fleig, Lena
Fleischhacker, Sheila
Fleming, Marc L.
Flemming, Hans-Curt
Flemming, Aysha
FLEURY, Marie José
Floros, Georgios
Fobil, Julius
Focsan, Alexandrina L.
Fogler, Kethera A.
Folwarczna, Joanna
Font, Laia
Foote, Janet A.
Foradori, Chad D.
Foraker, Randi E.
Forbes, Kenneth
Forbes, Professor Vivian
Ford, Casandra
Ford, Tim E.
Ford, Julian D.
Ford, Judy
Forjaz, Maria João
Fortier, Stephen C.
Fortin, Claude
Foster, Warren G.
Fourches, Denis
Fournier, Isabelle
Fowle, John
Fowmes, Gary J.
Fox, Chester H.
Fox, Aaron D.
Fox, Geoffrey
Fracgp, Georga Cooke
Fraedrich, Klaus
Francey, Thierry
Franco, Sandra
Franklin, Teresa R.
Frans, Van Geer
Frasch, H. Frederick
Frazer, Kate
Frazzoli, Chiara
Freiberger, Ellen
Freitas, Mariella B.
Frenzel, Wolfgang
Frew, Paula M.
Friesen, Melissa C
Friger, Michael
Friswell, Rena
Froelich, Wojciech
Froelich, Brett
Fröhlich, Jürg
Frontistis, Zacharias
Fry, Jillian P
Fu, Qiang
Fucikova, Anna
Fuermaie, Anselm BM
Fuertes, Elaine
Fuglsang, Katrine
Fujibe, Fumiaki
Fujimoto, Nariaki
Fujinaga, Aiichiro
Fujioka, Roger
Fujita, Misuzu
Fukuda, Daiju
Fukushima, Masami
Funtikova, Anna
Furin, Jennifer J.
Fyhri, Aslak
Gabriel, David
Gabriel, Patricia
Gaebel, Wolfgang
Gain, Animesh
Galanakis, Charis
Gallagher, John
Gallardo, Antonio
Gallas-Lindemann, Carmen
Gallego, Ángel
Galli, Gianfranco
Gallinat, Jürgen
Gallis, Christos
Gallus, Silvano
Galve, Roger
Gambardella, Chiara
Gamet-Payrastre, Laurence
Ganapati, Emel
Gangas, Carmen Gloria Ili
Gao, Xiang
Gao, Zhiye
Gao, Yan
Gao, Yang
Gao, Chuansi
Gao, Xubo
Garcés, Carmen
Garcia, Valerie
Garcia-Gasca, Silvia Alejandra
García-Rodríguez, Francisco J.
Gardiner, Philip
Garnick, Deborah
Garza-Rodríguez, Iliana de la
Gaskin, Darrell J.
Gaspar, Tania
Gassel, Margy
Gast, Richard K.
Gaudet, Pascale
Gazouli, Maria
Gebel, Thomas
Gebremariam, Seyoum Y.
Geer, Laura A.
Geiger, Sarah Dee
Geiger-Brown, Jeanne M.
Geist, Tracy S.
Gen, Sheldon
Genchi, Claudio
Gendall, Phil
Genna, Catherine
Gentry-Shields, Jennifer
Genuis, Stephen J.
Gerna, Giuseppe
Ghebremichael, Kebreab
Ghedin, Elodie
Ghosh, Rebecca
Ghosh, Apuma
Giacobbi, Peter
Giacomino, Agnese
Gianicolo, Emilio Antonio Luca
Giaouris, Efstathios
Gibb, Herman
Gibbons, Joseph
Gibney, R T Noel
Gibson, Kristen E.
Giesinger, Johannes
Gilbert, Kathleen
Gilca, Vladimir
Gillespie, Gordon Lee
Gilmore, Dave
Giloteaux, Ludovic
Gilroy, Ed
Giné Garriga, Ricard
Ginige, Athula
Girasek, Deborah C.
Giraud-Carrier, Christophe
Girones, Rosina
Gislason, Maya
Giuliani, Alessandro
Gladwell, Valerie
Glass, Kathryn
Glass, Nancy
Glass, Deborah C
Gobe, Glenda C
Goch, Aleksander
Godfroid, Jacques
Godycki-Cwirko, Maciek
Goel, Rajeev K
Goggins, William B.
Goh, Hong Eng
Gohlke, Julia M.
Goldberg, Lynette
Goldfinger, Judith Z.
Golding, Jean
Goldstein, Rachel Rosenberg
Goldstein, Sue
Golubnitschaja, Olga
Gomes, Patricia M.
Gómez de Tejada Romero, María Jesús
Gómez-López , Vicente M
Gomez-Rubio, Virgilio
Goncalves, Priscila
Gone, Joseph P.
Goñi, Pilar
Goniewicz, Maciej
González, María José
Gooch, Helen
Good, Kim
Goodman, Pat
Gorczynski, Paul
Gordon, Terry
Gordon, Gwyneth
Gorini, Francesca
Gosselin, Frédéric
Gould, Gillian
Graber, Judith
Grabowska, Ilona
Grace, Delia
Gradinger, Felix
Gragg, Richard
Graham, Jay
Granado, M. Dolores
Grant, Therese M.
Gray, Selena
Gray, Casey
Graziano, Joseph H.
Green, Jon
Green, Judith
Green, Jr., Harold D.
Greim, Helmut
Grenier, Patrick
Gressier, Florence
Grimes, Jack E. T.
Grindal, Grete Patil
Grogan, Sarah
Gromadzińska, Jolanta
Gronlund, Carina
Grönvall, Erik
Grossi, Giovanna
Gruber, Joshua
Grulich, Andrew E
Grundstein, Andrew J.
Grunig, Gabriele
Gualano, Maria Rosaria
Guauque-Olarte, Sandra
Guerrero-Preston, Rafael
Gugglberger, Lisa
Guglielmo, Riccardo
Guirguis, Kristen
Gulis, Gabriel
Gulisano, Massimo
Gulley, Tauna
Gulliver, John
Gulrud, Kristin
Gunatilake, Sarath
Günther, Sebastian
Guo, Ling-Zhong
Guo, Chaoran
Guran, Serpil
Gurley, Emily S.
Gushulak, Brian D.
Gustafson, Paul
Gustat, Jeanette
Gasana, Janvier
Guxens, Monica
Guzzi, Giampaolo
Gyouhyung, Kyung
Ha, Hoe-hun
Haaga, David A. F.
Haan, Mary N.
Haas, Jan
Habersaat, Stephanie
Haberstick, Brett C.
Haberzettl, Petra
Habuda-Stanic, Mirna
Hacker, Karen
Hackney, Madeleine
Haddad, Linda
Hajat, Anjum
Hak, Laura
Hales, Simon
Hall, Marissa G.
Hall, H. Irene
Hall, Myrna
Halonen, Jaana I.
Haluza, Daniela
Hamaguchi, Masahide
Hamano, Tsuyoshi
Hamdan, Motasem
Hamm, Michael
Hammig, Bart
Hammonds, Racheal
Han, Meekyung
Han, Xuemei
Han, Sang-Hwan
Han, Der-Sheng
Hanewinkel, Reiner
Hanibuchi, Tomoya
Hanigan, Ivan
Hanke, Wojtek
Hankey, Steve
Hanley, Sharon J.B.
Hansell, Anna L.
Hanson, Adrian
Hanzlíková, Hana
Haque, Reina
Haque, Ubydul
Hara-Kudo, Yukiko
Hardy, Kristina K.
Hare, Dominic J.
Harley, David
Harrigan, Ryan J.
Harrington, Kathleen F.
Harris, Shelley A.
Harrison, Flo
Hartig, Terry
Harzer, Claudia
Hassan, Sayed
Hassan Solórzano, Bernadette
Hassell, Kathryn L.
Hausmann, Michael
Havstad, Suzanne L.
Haw, Sally
He, Qinghua
He, Baokun
Hearst, Mary O.
Heath, Greg
Heaviside, Clare
Hebbert, Michael
Heckley, Gawain
Hedman, Ann-Marie Rydholm
Heffernan, Thomas
Hefferon, Kathleen Laura
Hegde, Muralidhar L.
Heim, Sabine
Heinemeyer, Gerhard
Heinonen, Ilkka
Heinonen-Tanski, Helvi
Heinrich, Angela
Heinrich, Joachim
Hellack, Bryan
Hemingway, Ann
Henderson, Claire
Henderson, J. Neil
Hendriks, Anna-Marie
Hendryx, Michael
Henkler, Frank
Hennessy, Emily
Hennig, Frauke
Henry, Sabine J. F.
Her, Moon
Herian, Mitchel
Hering, Alexandra
Herman, Katya
Hernandes, Rodrigo
Heroux, Paul
Herrera-Melián, José Alberto
Hesketh, Kathryn
Hesse, Morten
Hettiarachchi, Mala
Heukelbach, Jorg
Heus, Ronald
Heusser, Rolf
Hewlett, Sarah
Heywood, Anita
Hick, Rod
Higashino, Makoto
Higgins, Niall S
Hildenbrand, Georg Lars
Hill, Harvey
Hill, Jennie
Hillhouse, Joel
Hillstrom, Kathryn
Himmelgreen, David
Hinckson, Erica
Hine, Julian
Hines, Cynthia J.
Hinojosa, Melanie Sberna
Hintsa, Taina
Hipgrave, David B
Hirata, A.
Hirsch, Jana
Hiscock, Rosemary
Hiyoshi, Ayako
Hiza, Hazel A.B.
Hjorth, Peder
Hnatiuk, Jill
Ho, Sai Yin
Ho, Jeff C.
Hodgkinson, Stacy
Hodgson, Isaac O. A.
Hoeft, Theresa
Hoepner, Lori A.
Hoffmann, Markus
Hofman-Caris, Roberta
Hogan, Anthony
Holahan, Matthew
Holland, Stephen
Hollander, Guus de
Hollander, Petra Den
Hollen, Vera
Holmes, Dawn E
Holroyd, Eleanor
Holvoet, Paul
Homa, David M.
Hömme, Meike
Honda, Akiko
Hondula, David
Hong, Bongghi
Hong, Yun-Chul
Hongoh, Valerie
Hoogewerf, Arlene J.
Hoque, Mohammad A.
Hordacre, Brenton
Horner, Mark W
Hornikx, M.C.J.
Horsfall, Laura
Horst, Axel
Horton, Mark
Hosgood III, H. Dean
Hoshiko, Sumi
Hosomi, Naohisa
Hossain, Bakhtiar
Hossain, Moazzem
Hou, X Y
Houghton, Katherine
Hout, Joseph
Hovell, Melbourne
Hovem, Jens M.
Howard, John
Howden-Chapman, Philippa
Howell, Donelle N
Howell, Fenton
Howell, Andrew J.
Howie, Erin K.
Howieson, Stirling G.
Høye, Anne
Hrubá, Drahoslava
Hseu, Zeng-Yei
Hsia, Jason
Hsieh, Nan-Hung
Hsieh, Ping-Hung
Hsu, Kuo-Chin
Hsueh, Yu-Mei
Hu, Jie
Hu, Kaiwen
Hu, Anming
Hu, Xuefei
Hu, Hui
Hu, Liang
Hua, Xin
Huan, Guo
Huang, Zheng Y. X.
Huang, Guohe
Huang, Guangwei
Huang, Wu-jang
Huang, Yu
Huang, Cunrui
Huang, Wan-Ling
Huang, Yu-Tung
Huang, George Tzou-Chi
Huang, Yue
HUANG, GORDON
Hubbard, Katherine A.
Hübner, U.
Hudzik, Bartosz
Huedo-Medina, Tania B.
Huen, Karen
Huesca, Margarita
Huf, Samuel
Hung, Yung-Tse
Hunt, Piper Reid
Hurlbert, Marc
Hursthouse, Andrew
Hurtado, Ana
Hurtado, David
Hurwitz, Gil
Huss, Anke
Hwang, Jungho
Hynds, Paul D.
Iacobini, Carla
Iannuzzi, A.
Ibekwe, Mark
Ibrahim, Salam A.
Ichiba, Masayoshi
Idler, Ellen L.
Ifelebuegu, Augustine O
Igbinosa, Etinosa
Ihle, Andreas
Ikäheimo, Tiina
Ikem, Abua
Illsley, Matt
Imai, Cindy Mari
Imrie, Rob
Inman, Arpana G.
Innocent, Giles
Inostroza, Luis
Iossifidou, Eleni
Iriti, Marcello
Irvine, Ian
Isada, Tomonori
Isaksson, Anders
Isern, David
Ishaque, Ali B.
Isidori, Emanuele
Isidoro, Jorge M.G.P.
Islam, Farah
Issa, Touma B.
Issinger, Olaf-Georg
Istfan, Nawfal
Itani, Osamu
Ivanov, Ivan
Iweriebor, Benson
Izquierdo-Useros, Nuria
Jäckel, Udo
Jackson, Patricia
Jackson, Michael L.
Jackson, Laura
Jackson, Corrie L.
Jackson, Ronald
jacob, Lorenzo-Morales
Jacobs, David E.
Jacobsen, Sven-Erik
Jacquemin, Benedicte
Jaffrezic-Renault, Nicole
Jägerbrand, Annika
Jagger, Pamela
Jagnoor, Jagnoor
James, Philip
James, Margaret
James, William
James, Katherine A.
Jamil, Muhammad S
Janes, Victoria
Janssen, Sabine
Jansson, Märit
Javadi, Akbar
Jay, Jennifer A.
Jay, Sarah
Jenkins, Jill A.
Jensen, Carsten
Jeon, Byung-Hwa
Jernigan, Valarie Blue Bird
Jetter, James J.
Jia, Chunrong
Jia, Chongqi
Jia, Zhiyun
Jiang, Chengsheng
Jiang, Nan
Jiang, Hong
Jiang, Bin
Jien, Shih-Hao
Jin, Fan
Jing, Liang
Joergensen, Torben
Johannessen, Gro
Johler, Sophia
John, Kaarthik
Johnsen, Svein Åge Kjøs
Johnson, Laura
Johnson, Blair
Johnson, Donald R.
Johnston, Carl Gordon
Johnston, Jill E.
Johnston, Fay
Johnston, Jill
Jones, Tineke
Jones, Wendy
Jones, Lovell
Joossens, Luk
Jordan, Kieran
Jorgensen, Palle E.T.
Jose Manuel, Rodriguez-Llanes
Joseph, Wout
Juan, Y.K.
Juan Fernández, Aránzazu De
Juarez , Paul D.
Julander, Anneli
Julian, Tim
Jung, Dawoon
Jung, Jae Hee
Jurk, Diana
Jurzik, Lars
Jutla, Antarpreet S.
Jyh-fei, Liao
K, Skalska
Kaaya, Archileo Natigo
Kabir, Zubair
Kaczmarek, Lukasz D.
Kaczynski, Andrew T.
Kadam, Umesh
Kagan, Calvin M
Kagawa, Masaharu
Kagawa, Takahide
Kah, Melanie
Kahane, Rémi
Kahlmeier, Sonja
Kaiser, Anelis
Kalkstein, Adam
Källén, Bengt
Kallweit, Dagmar
Kamada, Masamitsu
Kamegawa, Takashi
Kamenov, George
Kamiński, Marcin
Kamouchi, Masahiro
Kane, Eleanor
Kaneko, Hideo
Kanerva, Noora
Kang, Choong-Min
Kang, Jaeku
Kannel, Prakash Raj
Kao, Chien-Ya
Kao, Jia-Horng
Kao, Kung-Ting
Kaplowitz, Stan A.
Kappell, Anthony
Kapusta, Nestor D.
Karabetsos, Efthymios
Karah, Nabil
Karakitsios, Spyros
Karanis, Panagiotis
Karayiannis, Peter
Karim, Salim Abdool
Karthe, D.
Karuppannan, Sadasivam
Kasabova, Mariana
Kashian, Donna R.
Kassamali, Zahra
Katbamna, Bharti
Kato, Ikuko
Katsogiannis, - Athanasios
Kauer, Sylvia
Kaufman, Jay
Kauko, Leiviskä
Kautsky, Ulrik
Kavanagh, John
Kavazanjian, Edward
Kavé, Gitit
Kedia, Satish
Keegan, Richard
Keenan, Rod
Kegler, Michelle
Keig, Peter
Keil, Alexander P
Kelepertzis, Efstratios
Keller, Melanie M.
Kempson, Ivan M.
Kennair, Leif Edward Ottesen
Keong, KWOH Chee
Kepka, Deanna L.
Kern, Janet
Kershaw, Kiarri N.
Kessler, Theresa A.
Khan, Izhar
Khan, Hafiz T. A.
Khan, M M
Kharma, Mohamed Yasser
Khashan, Ali S.
Khatiwada, Janak
Kheifets, Leeka
Khlifi, Rim
Kho, Hong-Seop
Khuder, Sadik
Kiem, Anthony
Kim, Hyeongsu
Kim, Yangho
Kim, Byungho
Kim, Hyun-Jun
Kim, Youngwook
Kim, Bumseok
Kim, Hyoung-Ryoul
Kim, Hyun
Kim, Jong-Suk
Kim, Jeungim
Kim, Kyounghee
Kim, Jong-Min
Kim, Sun-Young
Kim, Jung-Ha
Kim, Hyung Jin
Kim, Eun-Jung
Kimbrough, Sue
Kimpe, Norbert De
King, Mindy H.
Kinilakodi, Harisha
Kinney, Patrick
Kintzios, Spyridon
Kipen, Howard M.
Kirby, Joanna
Kirchengast, Sylvia
Kircher, Manfred
Kirkevold, Marit
Kirkland, Tabitha
Kishida, Naohiro
Kitahara, Cari M.
Kittles, Rick
Kittner, Jens M.
Klaperski, Sandra
Klaschka, Ursula
Klatt, Nichole R
Kleiman, Evan M.
Klein, Gunter
Klein, Bern
klimes-dougan, Bonnie
Klurfeld, David
Kluza, Jérome
Knapp, Martin
Knappett, Peter S.K.
Ko, Philip C.
Ko, Kyung-Seok
Ko, Ming-Sheng
Koch, Manja
Kodama, Keiichi
Koerner, Robert
Koga, Marius
Koh, Wan
Kohl, Harold W
Kohler, Jeffery
Kolivras, Korine N.
Kómar, Ladislav
Komiti, Angela
Komljenovic, Dragan
Komorowski, Jan
Kone, Karna Georges
Kong, Qiang
Kongar, Elif
König, Hans-Helmut
Konstantinou, George
Koo, Tebbin
Koo, Malcolm
Kopcakova, Jaroslava
Korhonen, Ilkka
Kortbeek, John
Korzeniewska, Ewa
Kosidou, Kyriaki
Kostner, G.M.
Kotulak-Balak, Malgorzata
Koukouzas, Nikolaos
Koulouglioti, Christina
Koupenova-Zamor, Milka
Koutrakis, Petros
Kovacic, Melinda S. Butsch
Kovesi, Tom
Kowalska, Małgorzata
Koyama, Asuka
Kozai, Naofumi
Kraemer, George P.
Kramer, Louisa
Krau, Stephen D.
Kraus, Ute
Kraut, Allen
Kravchenko, Julia
Krieg, Edward
Kripke, Clarissa
Krishnamoorthy, K.
Krishnamurthy, P.K.
Krishnan, Ranjini M.
Kroesen, Maarten
Kroll, Alexandra
Krometis, Leigh-Anne H.
Krop, Esmeralda
Krouse, Helene
Krysinska, Karolina
Kuan, Yu-Hsiang
Kuate-Defo, Barthélémy
Kubota, Naoto
Kucharski, Jan
Kulaga, Zbigniew
Kull, Inger
Kumar, Sajeesh
Kumar, Sumit R
Kumar, Sanjay
Kumar Ganguly, Nirmal
Kundi, Michael
Kunst, Anton
Kuntz, Benjamin
Kuo, Shu-lung
Kuo, Tony
Kuo, Chi-Chien
Kuo, Yi-Liang
Kupers, Terry
Kurisu, Kiyo
Kuyini-Abubakar, Ahmed Bawa
Kvítek, Libor
Kwan, Mei-Po
Kwok, Alison
Kwok Wai, Tham
Kyriacou, Charalambos P.
L. Melquiades, F.
Laane, Remi.W.P.M.
Laas, Alo
Labo, Nazzarena
Laborde, Sylvain
Labouliere, Christa D.
Ladas, Elena
Ladisa, G.
Lagergren, Mårten
Lagroye, Isabelle
Lai, Ka Man
Lai, Shih-Wei
Lai, Chuan-Yu
Lai, Gabriel
Lai, Peggy
Laidlaw, Mark
Laird, Eamon
Lalucat-Jo, Lluís
Lam, Benjamin Chih Chiang
Lam, Hubert
Lambertini, Elisabetta
Lambrou, Theofanis
Landau, Katheryn I.
Lang, Yinhai
Lang, Graeme
Lang, Jessica
Langdon, Kate A.
Langer, Sarker
Langergraber, Günter
Langford, Aisha T.
Langley, Tessa
Lapolla, A.
Lapworth, Dan
Lardies, Carlos Javierre
Larkin, Andrew
Lastella, Michele
Lauber, Christian L
Laumbach, Robert
Laverge, Jelle
Lavoie, Michel
Law, Chi-kin
Lawn, Sharon
Lawrence, Claire
Lawrence, David A.
Lawson, Justin
Lawson, Joshua A.
Laxe, Sara
Laxton, Adrian W.
Le Bot, Barbara
Leakey, Roger
Lean, David
Lebeis, Sarah L.
Ledda, Mario
Lee, Brain
Lee, Lucy E. J.
Lee, MiKyung
Lee, Joseph
Lee, Ming-Ju
Lee, Li-Yueh
Lee, Sang-Woo
Lee, Sun Y.
Lee, Sang-han
Lee, Fred
Lee, Juliana Tsz Yan
Lee, Hae K.
Lee, Jungeun Olivia
Lee, Joseph G. L.
Lee, Yau-Jiunn
Leffondré, Karen
Lefticariu, Liliana
Legros, Alexandre
Leh, M
Lehucher-Michel, Marie-Pascale
Lei, Zhongfang
Leidner, Andrew J.
Leith, David
Lenehan, Claire
Lenoble, V.
Lenoir, Irene
Leon, Maria E.
Leon, Lisa R.
León, Gerardo
Leonard, Nola
Leone, Antonio
Leong, David Tai
Leoni, Erica
Leotsinidis, Michalis
Leprevost, Catherine E.
Lequere, Antoine
Lerner, Chad
Leslie, Timothy F.
Leszczynska, Danuta
Leung, Ping-Chung
Leung, Ross Ka-Kit
Levantesi, Caterina
Levy, Ilan
Lewis, Ryan
Li, Wen-Whai
Li, Jian
Li, Xiujun
Li, Jun-wen
Li, Qingfeng
Li, Sijian
Li, Xuan
Li, Li
Li, Baoguang
Li, Tiantian
Li, Weijuan
Li, Peiyue
Li, Xian-Zhi
Li, Xinming
Li, Baizhan
Li, Siyue
Li, Shijian
Li, Linlin
Lian, Ie-Bin
Liang, Xiao-feng
Liao, Felix
Liao, Jiunn-wan
Liao, Yilan
Liao, Xianchun
Liao, Yung
Liao, Jing
Liapi, Charis
Lii, Tzu Wu
Liaw, Yung-Po
Licht, Andrea S
Lichter, Michal
Lichtveld, Maureen
Licínio, M.V.
Lien, Nanna
Lilenfeld, Prof. Lisa
Lilja, Margareta
Liller, Karen
Lim, Sungwoo
Lim, David
Lima-Filho, J.v.
Lin, Kwan-Hwa
Lin, Jin-Ding
Lin, John Z
Lin, Shinfeng D.
Lin, Chia-Her
Lin, Hanzhi
Lin, Pao-Hwa
Lin, Johnson
Lin, Yuan-Chung
Lindegård, Agneta
Lindkvist, Marie
Lindquist, Mark
Ling, Min-Pei
Liou, Peter
Liou, Der-Ming
Lipman, Len
Lippe, Roger
Litman, Tod
Litonjua, Ausgusto
Liu, An
Liu, Hongling
Liu, Haoyang Haven
Liu, Boning
Liu, Jingling
Liu, Shih-An
Liu, Wen-Cheng
Liu, Xiaoping
liu, guangxu
Liu, Li-Fan
Liu, Junjie
Liu-Ambrose, Teresa
Llewellyn, Gwynnyth
Lo, Kwong-Fai
Lobova, Tatiana I.
Locarnini, Stephen A.
Lodovici, Maura
Logan, Alan C.
Logue, Jennifer
Loke, Alice Yuen
Lollar, Don
Long, Thomas C.
Long, Christopher M.
Longmire-Avital, Buffie
Lopes, José Fortes
Lopez, Dina
López-Abán, Julio
Lopez-Garcia, Esther
Lopez-Sublet, Marilucy
Lora, America Miranda
Loretz, Linda
Lori, Valentina
Lou, Jie-Chung
Loukola, Anu
Loustalot, Fleetwood
Love, Tanzy
Lovreglio, Piero
Low, Chien-Tat
Low, Chien Tat
Lowe, Rachel
Lu, Jingrang
Lu, Weizhen
Lu, Weihong
Lu, Hongfang
Lu, Pengjun
Lu, Diannan
Lu, Geyu
Lucas, Françoise S.
Lucke, Terry
Lue, Hung-Chi
Lukkahatai, Nada
Lumen, Annie
Lumey, Lambert
Luna, Marcos
Lund, Sigrun Helga
Lunsky, Yona
Lunze, Karsten
Luo, Pingping
Luo, Ming
Luo, Bin
Luque, John S.
Luzzatto, Lucio
Lv, Jianshu
Lyle, David
Lyles, Courtney
Lynam, Mary
Lynch, Krystal
Lysklett, Olav Bjarne
Łuczkiewicz, Aneta
Łuszczyńska, Aleksandra
M. Lederer, Alyssa
Ma, Lu
Ma, Yuanmei
Ma, Chang-Xing
Ma, Yongjie
Ma, Hongbao
Ma, Guansheng
MacCalman, Laura
MacDonald, Morag
MacDonald Gibson, Jacqueline
Macfarlane, Duncan
Machtinger, Ronit
MacKenzie, Graham
MacMillan, Freya
MacNaughton, Neil S.
Macy, Jonathan T.
Madronich, Sasha
Madsen, A.M.
Magee, Christopher
Magin, Parker J.
Magliocco, Adriano
Magnani, Corrado
Magnavita, Nicola
Maia, José A. R.
Maibach, Edward
Mailey, Emily
Maisonet, Mildred
Malandrino, Mery
Malard, Véronique
Maldarelli, Frank
Maldonado, George
Malecki, Kristen
Malhotra, Tina
Malina, Robert
Malve, Olli
Mamudu, Hadii M.
Mancini, Monica
Mandelli, Laura
Mandic, Sandra
Mangal, Tara
Mangwandi, Chirangano
Maniecka-Bryla, Irena
Manjón, Guillermo
Mankey, Tina A.
Mannaerts, Chris
Mannucci, Pier Mannuccio
Mansfeldt, Tim
Mansoubi, Maedeh
Mansourian, Ali
Mantas, Vasco M
Mao, Liang
Maounounen-Laasri, Anna
Marc, Daniel
Marcheggiani, Stefania
Marchetti, Gregory F.
Margolis, Helene
Marian, Mary
Mariano, Ana Paula Melo
Marini, Martino
Marion, Jason W.
Marques, Alda
Marquis-Favre, Catherine
Marselle, Melissa
Marsh, Louise
Marshall, Alison L.
Marshall, Nadine
Marston, Louise
Martin, Faith
Martín, David
Martin, Loren A.
Martinasek, Mary
Martínez, Ramón Peña
Martinez, Lourdes
Martinez, Victor
Martinez Dominguez, Francisco
Martinez Lopez, Antonio
Martínez-Graña, Antonio
Martínez-Sánchez, Jose
Martinez-Urtaza, Jaime
Martinich, Jeremy
Martinotti, Giovanni
Martins, Maria
Martins, Fernando Gomes
Marván, Ma Luisa
Marzell, Miesha
Masala, Giovanna
Masaphy, Segula
Maschke, Christian
Maselko, Joanna
Maser‌, Edmund
Masho, Saba W.
Masoko, Peter
Mason, Lisa Reyes
Massányi, Peter
Massoni, Francesco
Masten, Susan
Matanda, Dennis J.
Mateus, Dina M.R.
Mathers, Amy J.
Matheson, Kimberly
Matias, Susana L.
Matranga, Domenica
Matsuoka, Yutaka
Matsuzawa, Yuji
Matthews, Charles E.
Mattson, Mats-Olof
Matzke, Marianne
Mavridou, Athena
Mavrogonatou, Eleni
Mawson, Anthony R
May, Philip
May, Warren L.
Maylor, Elizabeth
Mayner, Lidia
Mazumdar, Soumya
Mazumdea, Diya
Mazzanti, Andrea
McBride, Dawn M.
McBride, Graham
McCann, Clare
McCarthy, Mark
McCarty, Carolyn
McCawley, Michael
McCulloch, Kirsty
McDermott, Catherine
Mcdonald, Ted
Mcdonough, Suzanne
McDowell, Tiffany
McElroy, Peter
Mcgill, Anne-Thea
McGrail, Matthew M.
McGuire, Sarah
Mckee, David
Mckinstry, Brian
McLafferty, Sara
Mclain, Jean
McLaren, Lindsay
McLennan, Ian S.
Mcleroy, Ken
Mcmorrow, Tara
McMullin, Mary F.
Mcnally, Richard
McPhaul, Kathleen M.
Meadows, Graham N.
Medek, Danielle
Mehmood, Amber
Mei, Nan
Meiring, Tracy
Meiselman, Herbert L.
Mekary, Rania A.
Meléndez-Pastor, Ignacio
Melintescu, A.
Melnik, Bodo C.
Menai, Medhi
Méndez, Claudio A.
Meneghini, Elisabetta
Menke, Andreas
Menschikowski, Mario
Mere Del Aquila, Eduardo
Merecz-Kot, Dorota
Meredith-Jones, Kim
Mergler, Donna
Merianos, Ashley L.
Mericle, Amy
Merkx, Femke
Merrill, Jackie
Merson, Laura
Mesquita Mateus, Ricardo Filipe
Metzner, Jeffrey L.
Meyre, David
Michelozzi, Paola
Michels, Nathalie
Michielsen, Kristien
Midura, Ronald J.
Mikhailova, Elena
Milhausen, Robin R.
Millán Calenti, José C.
Miller, Mark
Miller, J. Mitchell
Miller, Kathleen E.
Miller, Charles D.
Millet, Maurice
Min-kyung, Lim
Miñarro, Marta Doval
Ming-Ping, Wu
Minich, Nancy A.
Minko, Tamara
Miranda, Regina Maura de
Miranda Jr., Robert
Mirauda, Domenica
Mirza, M. Monirul Q.
Mirzaei, Parham
Mita, Damiano Gustavo
Mitra, Sisir Kumar
Mitra, Raktim
Mitrou, Francis
Mitrović, Branislava M.
Mitsiade, Nicholas
Mittendorfer, Bettina
Miyamoto, Sheridan
Miyata, Yasuyoshi
Miyazaki, Yoshifumi
Mniszewski, Susan M.
Modesti, Pietro A.
Modin, Bitte
Moennikes, Oliver
Moffatt, Cameron
Moghe, Akshata
Moglia, Magnus
Mohnen, Sigrid M
Möhner, Matthias
Moliner-Urdiales, Diego
Molini, Patel
Moncada, Stefano
Mongkuo, Maurice Y.
Monroy, Oscar
Monsees, Thomas
Montaldo, Luisa
Montgomery, James
Montgomery, Hugh
Montuori, Paolo
Moodle, Indres
Moon, Hyun-Kyung
Moonesinghe, Ramal
Moore, Ginny
Moore, Roland
Moore, Kari
Moore, Richard Henry
Moore, Michael N.
Moore, Nathan
Morabito, Marco
Moraga-Serrano, Paula
Morean, Meghan Rabbitt
Moreau, John W.
Moreda-Piñeiro, Jorge
Moreno, Teresa
Moreno, Claudia
Moretti, Sandro
Moretto, Angelo
Morgan, Lloyd
Morgan, Richard K.
Mori, Jacopo
Moriarty, Elaine
Morici, Nuccia
Morin, Cory
Moriya, Shingo
Morland, Kimberly
Morley, Christopher P.
Morrell, Holly E. R.
Morrice, Emily
Morris, Vernon
Morris, Christopher
Morris, D S
Morris, Alison
Morris, David
Morris, Howard
Morris, Sheldon
Morrissey, Anne
Mortensen, Mary Ellen
Mortimer, Kevin
Moser, Thomas
Mosgöller, Wilhelm
Mosler, Hans-Joachim
Moss, Elica M.
Mou, Jin
Mouchtouri, Varvara A.
Moy, Ernest
MØLLER, Jens Kjølseth
Mudalige, Thilak K.
Mudrick, Nancy R.
Mufson, Laura
Muggah, Robert
Muhajarine, Nazeem
Müller, Bettina
Muller, Matthew D.
Muller, C.j.f.
Muller, Patricia A. J.
Mullins, Ben
Muñiz, Carlos Horacio Acosta
Munoz, Macarena
Munoz-Luna, Rosa Maria
Murakami, Keiko
Murakami, Michio
Murao, Satoshi
Murbach, Manuel
Murdoch, David R.
Murdock, Courtney
Murphy, Susan Kay
Murphy, Brendan
Murphy, Debra A.
Murphy, E.
Murphy, Alexia J.
Muscat, Joshua
Mustaquim, Moyen Mohammad
Mutowo, Mutsa
Myers, Christine Teeters
Myridakis, Antonis
Mzyaek, Fawaz
Nabhani, Shereen
Nachtigall, Paul E.
Naclerio, Gino
Nadais, M. Helena G.A.G.
Nadal, Martí
Nadal, Angel
Nahar, Vinayak
Nahvi, Shadi
Naidoo, Shan
Naik, Puneeta
Nair, Saraswathy
Nakano, Junta
Nakayama, Jiro
Nam, Dong-Ha
Namiesnik, Jacek
Napiórkowska-Krzebietke, Agnieszka
Narimatsu, Hiroto
Nastos, Panagiotis T.
Naughton, Geraldine
Nauman, Javaid
Navarrete-Muñoz, Eva Maria
Navarro, Ignacio
Navarro-Pedreño, Jose
Navas, José
Nawrocka, Agnieszka
Nebot, Carolina
Nekorchuk, Dawn
Nelson, Tony
Nemery, Benoit
Nemes, Szilard
Nemeth, Lynne
Neumann, Tim
Newman, John F.
Newton, Seth
Ng, Marie
Ng, Florrie
Ng, Jason
Ngai, Tommy
Ngom, Roland
Nguyen, Thin
Nicholls, Daniel
Nichols, Gregory P.
Nicol, Heather
Niedzielski, Przemysław
Niehof, Anke
Niehoff, Jens-Uwe
Niemeier, Janet
Nienaber, Cornelie
Niermann, Christina
Nieuwesteeg, Anke
Nieves, Kourtney
Nigro, Giovanni
Ning, Zhi
Nishihara, Eitaro
Nishimura, Izumi
Nishri, Ami
Niu, Jingpin
Nobuhiko, Tanaka
Nodine, Emily
Nofer, Jerzy-roch
Noller, Barry
Nonnemaker, James
Norman, Lisa
Norman, Trevor R.
Norr, Kathy
North, Michelle L.
Novack, Lena
Novoa, Stéfani
Novotny, Thomas E
Nowak, Glen
Nowalk, Mary Patricia
Nowicki, Michal
Nüesch-Inderbinen, Magdalena T.
Nugent, Rachel
Núñez Torres, María Isabel
Nuño, Roberto
Nyer, Maren
Nyirenda, Makandwe
Nymark, Penny
O'Brien, Marita
O'Donovan, Gary
O'mahony, Siobhain
O'shaughnessy, Patrick T.
O’mahony, Jim
O’Reilly, Ciara
Oberhelman, Sara
Ochiai, Tsuyoshi
Oddy, Wendy
Odigie, Kingsley O.
Odland, Jon Øyvind
Oesch, Franz
Ogawa, Wakano
Ohdo, Katsutoshi
Ojeda, Norma B.
Okafor, Maria-Theresa C.
Okafor, Florence
Okayama, Hiroto
Okayasu , Ryuichi
Okoli, Chizimuz
Oksa, Panu
Olaru, Doina
Oliveira, M. Beatriz P. P.
Oliveira, Carlos Augusto
Olivera, Pascual
Olivieri, Adam W.
Olsen, Jonathan
Olshansky, Stuart J.
Olson, Michael E.
Olson, James R.
Olson, Michael W.
Olver, Ian
Omumbo, Judy
Ong, Kwok Leung
Ongerth, Jerry
Onicescu, Georgiana
Onsted, Jeff
Onukwugha, Eberechukwu
Oosterkamp, Margreet
Op de Coul, Eline
Oppliger, Anne
Ordoñana, Juan R.
Oren, Eyal
Organelli, Emmanuelle
Orlando, Paolo
Ornelas-Soto, Nancy Edith
Oron, Gideon
Orosa, José A.
Orrell, Alison
Orrù, Germano
Osborne, Nicholas
Osganian, Stavroula
Ossip, Deborah J.
Osteen, Philip
Osterblom, Henrik
Osyczka, Anna Maria
Ota, Akinobu
Ott, Jördis
Ottosson, Jakob Ryd
Oursler, Merry Jo
Ouyang, Jinlong
Over, Eelco A. B.
Overland, Simon
Oviedo-Joekes, Eugenia
Owen, Daniel D.R.
Owens, Gary
Ownby, Dennis R.
Oxley, Tim
Oza-Frank, Reena B
Øverby, Nina C.
Paap, Kenneth R.
Paccaud, Fred
Pacheco, Joseph
Pachepsky, Yakov
Pacios, Luis
Paech, Gemma
Page, Timothy F.
Page, Wendy
Page, Anne-Laure
Page, Kristen
Palamar, Joseph J.
Palazzolo, Dominic L.
Palermo, Francesco
Palermo, Claire
Palm, Frederick
Palma, Carla
Palumbo, Carla
Pan, Xiaochuan
Pan, Gang
Panagopoulos, Thomas
Pancardo García, Pablo
Pandey, Abhishek
Pandey, Pramod K.
Pangbourne, Kate
Panic, Mirna
Panico, Salvatore
Pao, MARYLAND
Paoli, Greg
Papa, Maria Nicolina
Papadimitriou, Christina
Papadimitriou, Lampros
Papadopoulos, Vassilios
Papadopoulou, Crysanthi
Papas, Eric
Papazafiropoulou, Athanasia
Papi, Alessio
Papoutsakis, Constantina
Papp, András
Pappas, Steve
Parajuli, Rajendra Prasad
Paraskevas, Tsangaratos
Pardío-Sedas, Violeta T.
Parinandi, Narasimham L.
Paris, Marta
Parise, Carol A.
Park, Robert
Park, Jung-duck
Park, Miso
Park, Ki Hwan
Parker, Leslie A.
Parparov, Arkadi
Partonen, Timo
Paschall, Mallie
Pascua, Philippe Noriel Q.
Pashley, Catherine
Pasini, Margherita
Passy, Paul
Pastor-Valero, Maria
Patrick, Bob
Patterson, James W.
Patton, Allison
Paudyal, Vibhu
Pauly, Vanessa
Pavlíková, Daniela
Payne, Thomas J.
Payne, Sarah
Peacock, Oliver J.
Peacock, Amy
Pearce, Jo
Pearson, Jennifer L.
Pebsworth, Paula A.
Peck, Richard M.
Pedersen, Marie
Peeters, Carel F.W.
Pei, Yuansheng
Peijnenburg, Willie J. G. M.
Pekkinen, Minna
Peletz, Rachel
Pelicci, Pier G.
Pelkey, Neil
Peltier, Richard
Peña-Fernández, Antonio
Peng, Chien-fang
Peralta, Louisa
Pereira, Dolores
Pereira, Maria Do Carmo
Pereira, Cidália D
Peretz, Avi
Pérez, Sandra
Pérez, Glòria
Perez-Sanz, Araceli
Pergolizzi, Joseph V.
Perron, Stephane
Perrone, Maria
Perry, Lin
Persinger, Michael
Persson-Waye, Kerstin
Pesch, Beate
Peterson, Kristina
Petkova, Elisaveta
Petriciolet, A.Bonilla
Petrov, Megan E.
Petrovic, Daniel
Pettersson, David
Pettinen, Pasi
Pfaller, Stacy
Pfeffer, Karen
Phillips, Michael R
Phillips, Alison
Phillips, Karen
Phillips II, Gregory
Phung, Tri Dung
Pichini, Simona
Pickering, Amy
Pietrodangelo, Adriana
Pileggi, Silvana
Pimenta, Adriane M.
Piña, Benjami
Pintó, Rosa M.
Pires, José Carlos Magalhães
Piringer, Martin
Pirozzi, Cheryl S.
Pisarenko, Aleksey N.
Piscopo, Vincenzo
Pitkanen, Tarja
Pitt, Henry A.
Pizarro, Andreia
Pohl, Pawel
Pointer, Mildred A
Polimeni, Antonella
Polimeni, John M.
Polo-Kantola, Päivi
Polosa, Riccardo
Polya, David
Polzin, Steven
Pongpiachan, Siwatt
Popovici, Ioana
Portegijs, Erja
Portier, Kenneth M.
Post, Ernest M.
Pottee, Kevin
Potter, Julia
Poulin, Michel Bigras
Poulsen, Otto Melchior
Poulsen, Michael
Pouponneau, Pierre
Powell, Charles
Powell, John
Poynter, Jenny
Poynton, Timothy A.
Pradhanang, Soni M.
Prado, Gustavo Faibischew
Prasad, Vinayak
Preiser, Rika
Preve, Nikolaos P.
Prevots, Rebecca
Pribadi, Didit Okta
Price, Rumi Kato
Price, Polly J.
Prieto, Miguel
Prins, Gail S.
Prins, Marijn A.
Pritsos, Chris
Prochaska, John D.
Prochaska, Judith
Prodinger, Birgit
Prohaska, Xariklia
Provenzano, Maurizio
Prus, Steven
Przepiorka, Aneta M.
Przybysz, A.
Psaroulaki, Anna
Psarra, Anna-maria G.
Puddu, Paolo Emilio
Puett, Robin
Pullanikkatil, Deepa
Pumpa, Kate
Pup, Lino Del
Puzyn, Tomasz
Pyrgiotakis, Georgios
Qi, Feng
Qin, Yanwen
Qin, Hua-Peng
Qin, Zhanfen
Qiu, Yanling
Qiu, Yong
Quinn, Nigel W.T.
Quiñones, Beatriz
Quitmann, Julia Hannah
Qvist, Ina
R. Carter, Stephen
Raanaas, Ruth K.
Rabbitt, Sarah M.
Rachaniotis, Nikolaos Panagiotis
Rafalson, Lisa
Raheem, Adeeba A.
Rahman, Khaja Zillur
Rahmberg, Magnus
Rainisch, Bethany
Raitasalo, Kirsimarja
Rajaee, Mozhgon
Rajagopalan, Priya
Rako, Nikolina
Ralston, Margaret L.
Ramesh, Aramandla
Ramírez, M. S.
Ramirez Harrington, Donna
Ramos, Diana E.
Ramos, Jose Manuel
Randall, Ray
Randall-Simpson, Janis
Rantakokko, Merja
Rapheal, Benny
Raskin, Lutgarde
Rasmussen, Jon B.
Ratcliffe, Eleanor
Ratick, Samuel J.
Ravegnini, Gloria
Raven, Melissa
Raymer, James H.
Raynal, Anne
Razum, Oliver
Rediske, Richard R.
Redsteer, Margaret Hiza
Reed, Maureen G.
Reed, Justy
Reeves, Roger Keith
Rehman, Laurene
Reichmayr, Johannes
Reichwaldt, Elke
Reidenbach, Hans-Dieter
Rein, David B.
Reinhardt, Jan Dietrich
Reinhardt, Jan D.
Reinier, Kyndaron
Reis, Flávio
Reisen, William K.
Reiss, Richard
Reiss, Kelly Chinners
Reissmann, Daniel R.
Renato, Iannelli
Renzetti, Claire M.
Repace, James
Reppermund, Simone
Resnik, David B.
Reszka, Edyta
Rey-Rico, Ana
Reyes, Teófilo
Reyes-Gómez, Víctor M.
Reynolds, Amy
Rhodes, Glenn
Ribisl, Kurt
Riccardo, Flavia
Riccio, Angelo
Rice, Penelope A.
Richards, Dan
Richmond, Janet
Richmond-Bryant, Jennifer
Riddell, Lynn
Rider, Cynthia
Riedel, Natalie
Riedel, Philipp
Riediker, Michael
Rieffe, Carolien
Rietbergen, Martijn G.
Rietra, Rene
Rikard, R.V.
Rimondini, Lia
Rioux, Christine L.
Riskind, Rachel G.
Riskowski, Jody L.
Risser, Ralf
Rivadulla, Casto
Rizzica, Lucia
Ro, Annie
Roberts, Adam
Roberts, Sheila J.
Roberts, Megan
Roberts, Megan C.
Robertson, Deborah L
Robertson, Lucy
Robertson, Suzanne
Robey, Kenneth L.
Robinson, Guy
Robson, Mark Gregory
Roca Pascual, Núria
Rocha, Joao B.T.
Rocheleau, Carissa
Rocklöv, Joacim
Rodea-Palomares, Ismael Martin
Rodrigues, António S.
Rodrigues, Jean-Marie
Rodriguez Martin, Jose Antonio
Rodríguez-Couto, Susana
Rodríguez-Fórtiz, María José
Rodríguez-Lado, Luis
Rodríguez-Martínez, Sara
Rodziewicz, Joanna
Roebuck, Bill
Roeter, Stephanie
Rogers, Ann E.
Rohr, Annette C.
Rohsenow, Damaris
Roig-Navarro, Antoni Francesc
Rojas-Rueda, D.
Romagna, Giorgio
Romagnoli, Costantino
Romalde, Jesús López
Romano, Stefania
Romer, Dan
Rönnlund, Michael
Rooks, Ronica N.
Roos, Anneclaire De
Rosário, Rafaela
Rosas, Scott R.
Rosen, Barry
Rosen, Laura
Rosenberger, Jaroslav
Rosenkranz, Richard R.
Rosenthal , Dean S
Ross, Craig
Rossi, Federico
Rössner Jr., Pavel
Rostkowski, Pawel
Roult, Romain
Rounds, Stewart
Rousu, Matthew
Routen, Ash C.
Rowe, Gene
Rowling, Louise
Roy-Engel, Astrid M.
Ruano-Ravina, Alberto
Rubio-Romero, Juan Carlos
Ruchter, Nadine
Ruden, Douglas
Ruhl, Aki Sebastian
Ruiz, Marilyn O
Ruiz, Tomás
Ruiz-Rodríguez, Juan C.
Ruiz-Ruiz, Carmen
Rumchev, Krassi
Rundle, Andrew
Russell, Kelly
Rutten, Erica
Ryadnov, Max
Ryan, Michael
Ryan, David K.
Ryan, Joseph
Ryckman, Kelli K.
Rylander, Ragnar
S. J. Tol, Richard
Saari, Rebecca K.
Sabbah, Wael
Sacco, Gail Dana
Sacks-Davis, Rachel
Sadanaga, Tsuneaki
Sadat, Jasmin
Sadatsafavi, Hessam
Saddleson, Megan
Sadler, Richard
Saez, Marc
Saha, Sanjib
Saikawa, Eri
Saint - Maurice, P e d r o F .
Sakamoto, Shuichi
Sakan, Sanja M.
Saladini, Francesco
Salas, Juan José
Salazar, Rodrigo Fernando dos Santos
Salgado, Cassandra D.
Salinas, Carla
Salloum, Ramzi G.
Samant, Yogindra
Samant, Tanay S
Samarasundera, Edgar
Sampers, Imca
Sanborn, Margaret
Sánchez, José Luis Reyes
Sánchez-Soberón, Francisco
Sandallı, Cemal
Sándr, Attila D
SANGWAL, Keshra
Sanmiquel Pera, Lluis
Sannibale, Claudia
Santamouris, Mat
Santantonio, Teresa
Santibanez, Tammy A.
Santoro, Luca
Santos, Paulo
Santos-Gallego, Carlos
Sargent, Alex
Sarigiannis, Dimosthenis A.
Sarubbo, Dr. Leonie A.
Sasaki, K.
Sass, Jennifer
Sato, Kuniaki
Satoh, Hisashi
Sauer, Ann Goding
Saulsbury, Marilyn
Savopoulos, Christos
Saxon, Andrew J.
Sayed, Nudrat Z
Sazma, Matthew
Sbihi, Hind
Scaletta, Giuseppe
Schaffer, Andrea
Schall, Carol M.
Schantz, Peter
Schatz, Enid
Schaubroeck, Thomas
Schauss, Alexander G.
Schebb, Nils Helge
Scheers, Hans
Scheffran, Jürgen
Scheid, Patrick
Scher, Deanna
Schewe, Rebecca L.
Schijven, Jack F.
Schikowski, Tamara
Schimmenti, Adriano
Schindler, Stefan
Schmid, Daniela
Schmidt, Morten
Schmidt, Jesper Hvass
Schmidt, Cheryl K.
Schmidt, Caroline
Schmiedeler, Sandra
Schmitt, François G.
Schmitz, Gerd
Schnatter, Robert
Schneider, Raphaël
Schnell, Izhak
Schobersberger, Wolfgang
Schoenbach, Victor
Schoeny, Rita
Schoeppe, Stephanie
Schreiber, Angélica Zaninelli
Schreinemachers, Dina
Schuchardt, Sven
Schuermann, David
Schulz, Peter J.
Schütte, Martin
Schutte, Anne
Schütze, André
Schwaiger, Karin
Schwartz, Jennifer
Schwartz, Robert P.
Schwebel, David
Schweizer, Pia-Johanna
Scobie, Linda
Scognamiglio, Viviana
Scortichini, Matteo
Scott, Theresa L.
Scott, Sam
Scrine, Clair
Scuffham, Paul
Seabra, Amedea B.
Searl, Allison
Searle, Amelia K.
Sedig, Kamran
See, Lai-Chu
Seeman, Mary V.
Seena, Sahadevan
Seghers, Jan
Segovia-Siapco, Gina
Segura-Egea, Juan José
Segura-Ortí, Eva
Seifert, John G.
Seixas, Noah S.
Seki, Katsutoshi
Sell, Katie
Sellamuthu, Balasubramanian
Selle, Benny
Sellix, Michael
Selmin, Ornella
Sembajwe, Grace
Semenzin, Elena
Sen, Souvik
Sendra, Esther
Sergeev, Alexander
Serra, Jordi
Servidio, Rocco
Sessa, Salvatore
Settanni, Luca
Shahabfar, Alireza
Shandro, Janis
Shang, Ce
Shao, Lei
Shao, Chaofeng
Sharkey, Alyssa B.
Sharma, Prabhakar
Sharpe, Richard
Sharpe, Tim
Sheehan, Karen M
Sheffield, Perry
Shen, Chien-wen
Sheng, Lianxi
Shepherd, Daniel
Shepherd, Anthony
Shi, Xianglin
Shield, Jenny
Shields, Vonnie
Shiffman, Saul
Shih, Patrick C.
Shimizu, Yuta
Shin, Jung-hye
Shin, Hai-Rim
Shoben, Abigail B.
Shoda, Makoto
Shoenfeld, Yehuda
Shoji, Yasushi
Shortt, Niamh K.
Shrestha, Surendra
Shridhar, Varsha
Shu, Hung-Yee
Siahpush, Mohammad
Sibanda, Timothy
Siebenhofer, Matthäus
Siegel, Robert
Siehl, Agemar
Sigmund, Erik
Signal, T. Leigh
Sikorski, Johannes
Silbergeld, Ellen
Silva, Adrián M.T.
Silverberg, Donald S.
Simeone, Regina
Simm, Roger
Simon, Stacey
Simon, Edina
Sims, Jane
Sims-Gould, Joanie
Sinclair, Ryan
Singh, Seema
Singh, Anita
Singh, Kameshwar P.
Singh, Tushar
Singhrao, Sim K.
Sinha, Satyesh
Sironi, Selena
Sit, Cindy Hui-Ping
Sixsmith, Judith
Sjöström, Rita
Skinner, Elizabeth H.
Skouteris, George
Škrbić, Biljana
Skuterud, Mikal
Skwarzec, Bogdan
Skytte, Lilian
Sladkova, Jana
Slowikowska-Hilczer, Jolanta
Smani, Younes
Smarr, Karen L.
Smarr, Cory-Ann
Smetacek, Victor
Smit, Lidwien
Smith, George Davey
Smith, Gordon
Smith, Brett
Smith, ANDRÉA LIVI
Smith, Timothy M.
Smith, Selina A.
Smith, Gillian
Smits, Helen
Snyder, Lynne Page
Sobral, Rita G.
Söderqvist, Fredrik
Soenen, Stijn
Sofer, Tamar
Soga, Masashi
Sojic, Aleksandra
Sokolova, Ekaterina
Solo-Gabriele, Helena
Solovyev, Nikolay
Sone, Hideko
Sonenberg, Andrea
Song, Xiaobo
Song, Lixin
Song, Peter
Soopramanien, Anba
Soors, Werner
Sophronides, Panayiotis
Sorahan, Tom
Sosa, Erica T.
Sotgiu, Giovanni
Soto, Natacha Aguilar de
Sousa, Nelson
Sousa, Mário
Souza, Sofia
Soylak, Mustafa
Soyseth, Vidar
Spaeth, Andrea M.
Spector, Denise
Speer, Paul W.
Spence, Sue
Spence, J. David
Spencer, Michael
Spetter, Carla
Spickett, Jeffery
Spiegel, Sam
Spilak, Michal Proctor
Spinney, Jamie
Squadrone, Stefania
Sreekanth, Janardhanan
Ssematimba, Amos
St. Hilaire, Melissa A.
Stabin, Michael G.
Stack, Steven
Stahl Jr., Ralph G.
Stallones, Lorann
Stalsberg, Helge
Stam, Rianne
Stamatakis, Michail
Stamatakis, Emmanuel
Stamatelos, Spyros K.
Stamatis, Nikolaos
Stamey, James D.
Stanhewicz, Anna E.
Staniscia, Tommaso
Stansfield, Kirstie
Stasi, Leandro L. Di
States, J. Christopher
Staufer, Katharina
Stawarz, Robert
Steenland, Kyle
Steer, J.
Stefanidou, Maria E.
Steinborn, Dirk
Steinman, Howard M.
Steinmaus, Craigm
Stevens, Chris
Stevens, Adam
Stevenson, Paul
Stewart, Susan L.
Stewart, Ian
Stewart, Scott H.
Stichnothe, Heinz
Stickley, Andrew
Stieb, David M.
Stilianakis, Nikolaos I.
Stinchcomb, David
Stingelin, Nicola
Stingone, Jeanette
Stocco, Carlos
Stockwell, Virginia
Stodden, David
Stoevesandt, Bernhard
Stokes, Hatch
Stoklosa, Michal
Stone, Vicki
Story, Michael
Stott, Tim
Stoupel, Eliyahu
Stoy, Paul
Strauser, David
Striley, Catherine W.
Strobel, Natalie
Stronks, Karien
Stuart, Venetia
Stubblefield, Andrew
Stylianou, Katerina
Su, Wen-Da
Su, Chunming
Su, Chang
Su, Meirong
Suárez-Estrella, F.
Suarez-Varela, Maria Morales
Subbaraman, Ramnath
Subramaniam, Mythily
Sucharew, Heidi
Suedel, Burton C.
Sujarwoto, Sujarwoto
Sullivan, William
Sumbele, Irene UN
Sumner, Walton Ii
Sun, Wenjie
Sun, Tao
Sun, Danfeng
Sunakawa, Yu
Sunasee, Rajesh
Sundin, George
Sundseth, Kyrre
Suñer-Soler, Rosa
Sung, Jidong
Suominen, Anna Liisa
Sureda, Xisca
Sussan, Thomas
Suter, Melissa A
Sutton-Grier, Ariana
Suwazono, Yasushi
Suzuki, Keiko
Svartengren, Magnus
Svoboda, Zdenek
Swanson, John
Swartz, James A.
Świątkowska, Beata
Syed-Abdul, Shabbir
Szyszkowicz, Mieczysław
Tabb, Loni Philip
Tabuchi, Takahiro
Taff, Derrick
Takamura, Noboru
Takebayashi, Hideki
Takeuchi, Masaru
Takkouche, Bahi
Talati, Megha
Talbert-Slagle, Kristina
Talbot, Prue
Talbott, Evelyn O.
Talhout, Reinskje
Tamaki, Naofumi
Tamerius, James D.
Tamura, Toshiyo
Tan, Xuhui
Tandy, Susan
Tang, Niansheng
Tang, Song
Tang, Tiantian
Tangari, Andrea
Tanida, Takashi
Taniguchi, Ayako
Tao, Yan
Tao, Shasha
Tao, Hong
Tappia, Paramjit S.
Tarolli, Paolo
Tasat, Deborah R.
Tassiopoulos, Katherine
Tawia, Susan
Taxvig, Camilla
Taylor, Caroline
Taylor, Andrea Faber
Taylor, Joe
Taylor, Julia A.
Taylor, Nigel A.S.
Tchicaya, Anastase
Teegavarapu, Ramesh S.V.
Teeguarden, Justin G
Temple, Norman J.
Tenailleau, Quentin M.
Teodoro, Cordova-Fraga
Terashima, Rafael Ponce
Terry, Patricia
Terry, Daniel
Teschke, Rolf
Teschner, Naama
Thackeray, Rosemary
Thakore, Sonal
Theorell, Töres
Thiagarajan, Thippi
Thielke, Stephen
Thomas, Roger E.
Thomas, David P.
Thomas, Stuart
Thomas, Evan
Thompson, Lisa
Thompson, Marcella Remer
Thompson, Wendy
Thompson-Paul, Angela
Thomson, Jessica L.
Thornton, Lukar
Thurston, George
Tian, Ying
Tibbetts, Stephen G.
Tigelaar, Dineke
Timberlake, David
Timiryasova, Tatyana M.
Timmermans, Harry
Titze, Sylvia
Tkac, Jan
Tobias, Jim
Tobiszewski, Marek
Toda, Kei
Todd, Ewen
Todem, David
Todorova, Svetoslava
Tokonami, Shinji
Tolley, Elizabeth A.
Tomoda, Fumihiro
Tomza-Marcinak, Agnieszka
Tondeur, Francois
Tong, Haiyan
Tong, Tiejun
Toomey, Clodagh
Torabi, Mahmoud
Torgersen, Geir
Torija, Antonio J.
Torres, Vaamonde Jose Enrique
Tositti, Laura
Townley, Greg
Townsend, Julie
Townshend, Tim
Toy, Mehlika
Trasande, Leonardo
Trask, Catherine
Trepka, Mary Jo
Trevisani, M.
Trujillo, Leonard
Trulli, Ettore
Trunk, Dunja
Tryland, Ingun
Tsai, Ying I.
Tsai, Chen-Chi
Tsai, Fuu-jen
Tse, La
Tseng, Tung-Sung
Tseng, Kuo-Hsin
Tsourtos, George
Tsuge, Masataka
Tsuji, Joyce
Tsumune, Daisuke
Tsuzuki, Yoshiaki
Tu, Jun
Tucker, Patricia
Tucker, Andrew
Tudor, Tl
turci, Francesco
Türk, Jochen
Turner, Lyle
Tyagi, Vinay Kumar
Tzivian, Lilian
Uchikoshi, Yuuko
Ueda, Kayo
Uehleke, Reinhard
Uejio, Christopher
Ukai, Takashi
Ullah, Aman
Unami, Koichi
Underwood, Lee A.
Urban, Aleš
Usher, Shane
Utay, Netanya Sandler
Uusi-Rasi, Kirsti
Uyttendaele, Mieke
Vaccarella, Salvatore
Vagedes, Jan
Vaiano, Vincenzo
Vakulin, Andrew
Valavanidis, Athanasios
Valdramidis, Vasilis P.
Vale, Diama
Valentukeviciene, M
Valenzuela, Hector
Valery, Patricia C
Vallini, Giovanni
van Acker, Veronique
Van Baak, Marleen A.
Van Bogaert, Peter
Van Boven, Job FM
Van Bruggen, Ariena
Van Cauwenberg, Jelle
van den Berg, Pauline
Van Den Bosch, Matilda
Van Den Brink, Nico
van den Driesche, Sander
Van Der Duin, Patrick
Van Der Linden, Sander
Van Hoof, Joris J.
Van Kamp, Irene
Van Raaij, Joop
Van Rees, Charles
van Rienenb, Ursula
Van Rongen, Eric
Vandermael, Alain
VanDerslice, Jim
VanGarsse, Anne
Vanos, Jennifer
Vaquera, Gloria S.
Varani, Luca
Várbiró, Gábor
Varouchakis, Emmanouil
Vasiliadis, Helen-Maria
Vasquez, Alejandro
Vaz Moreira, Ivone
Vedal, Sverre
Vedøy, Tord Finne
Veeranki, Phani
Veiga, Marcello
Velleman, Yael
Velly, Ana Míriam
Velten, Julia
Venieri, Danae
Venugopal, Jayarama Reddy
Vera, Luisa
Verhaak, Peter F.M.
Verhoeff, Arnoud
Verhoughstraete, Marc
Verlicchi, Paola
Verropoulou, Georgia
Vézina, Nicole
Viazzi, Francesca
Viegas, Carla
Viegas, Susana
Vieira, Edgar Ramos
Vigeh, Mohsen
Vighi, Marco
Vignozzi, Linda
Viguié, Catherine
Vijaykrishna, Dhanasekaran
Vilavert, Lolita
Vili, Nosa
Villalobos, Alice
Villalonga-Olives, Ester
Villarreal, Miguel
Vincent, Robert K.
Virani, Shama
Visai, Livia
Viscor, Gines
Vishnu, Abhishek
Vitali, Matteo
Vitas, Anabel
Vittori Antisari, Livia
Vlachantoni, Athina
Vlcek, Jiri
Vogt, Bruno
Vos, Theo
Vosvick, Mark A.
Voulvoulis, Nikolaos
Vrieling, Alina
Vriesekoop, Frank
Vymazal, Jan
Wachowiak, Gabriela
Waddell, Emmanuel
Wagiran, Husin
Wahed, Ahmed Abd El
Wainfan, Lynne
Waite-Cusic, Joy G.
Walker, Jimmy
Walker, Blake Byron
Wallace, Danielle
Wallentin, Gudrun
Walser, Sandra M
Walter, Michael H.
Walters, Matthew
Walters, Dianne
Walton, Kevin
Walton, AnnMarie
Walton-Roberts, Margaret
Walz, Roger
Wang, Yi
Wang, Harry HX
Wang, Hong
Wang, Yuankun
Wang, Zhenzhen
WANG, SHENGRUI
Wang, Wei
Wang, Shr-Jie
Wang, Peng-Hui
Wang, Wenyu
Wang, Chong-wen
Wang, Quanrong
Wang, Yan
Wang, Jianli
Wang, Lian-Sheng
Wang, Yundong
Wanner, Miriam
Warber, Sara Lesley
Ward, Mary
Ward, James
Ward, Doriel
Wardrop, Nicola
Wargocki, Pawel
Warner, Dorothy
Warren-Jeanpiere, Lari
Warshawsky, Daniel Novik
Washington, Alex
Waterman, Jenora T.
Watson, Michael C.
Watson, Alan
Watson, Silvana Maria Russo
Watt, John
Watts, Michael
Waugh, William
Waygood, Owen
Webb, Elizabeth
Webb, Dr. Thomas
Weber, Germain
Weber, Delene
Weber, Roland
Webster, Noah J.
Weg, Mark Vander
Wegener, Sandra
Wegner, Lisa
Wei, Binnian
Wei, Gan
Wei, Ran
Weidhaas, Jennifer
Weigel, Margaret
Weijs, Liesbeth
Weinmann, Tobias
Weinstein, Sally
Weinstein, Philip
Weishaar, Heide
Weitkamp, Alexandra
Weitschek, Emanuel
Welch, David
Wells, Ellen M.
Weng, Lu-Chen
Wenguo, Weng
West, Jane
Westaway, K.
Wettstein, Markus
Weyermann, Maria
While, Alison E.
Whiley, Harriet
White, Richard
White, Paul
White, Jason
White, Kim
Whitehead, Todd
Whitford, Heather
Whitsel, Laurie
Wiart, Joe
Wichman, Janine
Wicker, Pamela
Wickliffe, Jeffrey
Wieczorowska-Tobis, Katarzyna
Wieland, G. Daryl
Wiener, Judith
Wijnen, V.J.M.
Wiley, Kerrie
Wilker, Elissa
Wilkes, Lesley
Wilkinson, Kevin
Wilks, Martin
Willems, Sara
Williams, Brent J.
Williams, Craig
Williams, Susan
Williams, Rebecca
Williams, Adina
Williams, Kevin M.
Williams, David R.
Willie, Christopher K
Willis, Anusuya
Wilson, Josephine
Wilson, Leigh
Wilson, Hannah J.
Wim, Vandenberghe
Windham, Gayle C.
Windsor, L. Jack
Winefield, Tony
Winkler, Juergen
Winter, Jennifer
Winters, Carena
Wipfli, Heather
Wirtz, Philip
Wittmann, Marc
Wittrant, Yohann
Woith, Wendy
Wojtyczka, Robert D.
Wolf, Tanja
Won, Sungho
Wong, Grace Lai–Hung
Woo, Jessica Graus
Woo, Jong Min
Wood, Brittany S.
Woodcock, James
Woodfield, Lorayne
Woods, Alisa G.
Wooten, Joshua S.
Wordlaw-Stinson, LaShawn
Wreford, Anita
Wu, Kevin Chien-Chang
Wu, Jianyong
Wu, Jilei
Wu, Lingling
Wu, Kangsheng
Wu, Chin-Pyng
Wyles, Kayleigh
Wyver, Shirley
Xia, Chaoxiong (Michelle)
Xia, Yankai
Xia, Jihong
Xiang, Huiyun
Xiang, Hao
Xiao, Jianguo (Alex)
Xiao, Yonghong
Xiao Ying, Zheng
Xie, Yiqiong
Xie, Bangchang
Xu, Xiaobin
Xu, Linyu
Xu, Iris
Xu, Fenglian
Xu, Nan
Xu, Xiaohui
Xu, Yiqing
Xu, Qingwen
Xu, Jun
Yach, Derek
Yaghjyan, Lusine
Yahya, Al-ethawi
Yakub, Mohsin
Yakusheva, Olga
Yamada, Masakazu
Yan, Lijing
Yan, Weili
Yang, Yizhao
Yang, Yang
Yang, Xiaolin
Yang, Ce
Yang, Qingchun
Yang, Cheryl C.H.
Yang, Tie-Lin
Yang, Linsheng
Yanos, Philip T.
Yao, Sheldon
Yao, Yan
Yard, Ellen
Yarmand, Hamed
Yau, Matthew
Ye, Yun
Yeo, Yoon
Yim, Kyeong Sook
Yim, Un Hyuk
Yin, Jun
Ying, Guishuang
Yoder, Matthew
Yokota, Renata T.C.
Yokum, Sonja
Yoon, Seung Chul
Yorgason, Jordan
Yoshinaga, Jun
You, Zhiying
You, Hong
Young, April M.
Young-Rojanschi, Candice
Yu, Shaw L.
Yu, Peichao
Yu, Chuanhua
Yu, Kai
Yu, Zhisheng
Yu, Shu-Qin
Yu, Dapeng
Yu, Vivian
Yuan, Zuyi
Yuan, Zhen
Yuen, John Wm
Yuichi, Hyakawa
Yun, Dai
Yusuf, Farhat
Zaborskis, Apolinaras
Zaccarelli, Mauro
Zacchini, Massimo
Zalejska-Jonsson, Agnieszka
Zaluar, Alba
Zamaratskaia, Galia
Zeiner, Michaela
Zema, Demetrio Antonio
Zeng, Guangmin
Zeng, Di
Zeugolis, Dimitrios
Zhang, Jinliang
Zhang, Huiping
Zhang, Qianyi
Zhang, Kai
Zhang, Guanghao
Zhang, Lei
Zhang, Lang
Zhang, Aiyong
Zhang, Haibo
Zhang, Lufa
Zhang, Xiaotong
Zhang, Jianying
Zhang, Susan
Zhang, Yanyan
Zhang, Yonghong
Zhao, Jiajun
Zhao, Bin
Zhao, Fang-Hui
Zhao, Kun
Zheng, Hailei
Zhong, Weihong
Zhou, Shang-Ming
Zhou, Xiaonong
Zhou, Yang
Zhou, Xuan
Zhou, Zhijun
Zhu, Hong
Zhu, Kangmin
Zhu, Guangwei
Ziebarth, Nicolas R.
Zoladz, Phillip R.
Źróbek-Sokolnik, Anna
Zucker, Donna
Zutphen, Alissa Van
Zwahlen, Marcel

